# International comparison of patient care trajectories: Insights from the ICCONIC project

**DOI:** 10.1111/1475-6773.13887

**Published:** 2021-11-10

**Authors:** Irene Papanicolas, Jose F. Figueroa

**Affiliations:** ^1^ Department of Health Policy London School of Economics London UK; ^2^ Department of Health Policy and Management Harvard T.H. Chan School of Public Health Boston Massachusetts USA

## 1 INTRODUCTION

Health systems across high‐income countries have similar goals, which include maximizing quality of care, offering services responsive to patient needs, and ensuring efficient health care delivery.^1^, ^2^ Health systems also face similar challenges, such as changing demographics, limited national resources, and ongoing rising health care costs.^3^ In response, national policy makers are working to identify effective strategies to address these challenges, which are heavily influenced by existing health system features. A group of particular concern is the growing number of high‐need, high‐cost (HNHC) patients,^4^ a clinically diverse set of patients with multiple medical needs, frailty, and multimorbidity.^5^, ^6^ While constituting a relatively small proportion of the population, these patients account for a disproportionate share of medical expenditures across health systems.^7^, ^8^, ^9^, ^10^

International comparisons of patient care trajectories have the potential to provide policy makers and clinical leaders with valuable insights into how the organization of care delivery influences the effectiveness and efficiency of the care they provide. However, despite the critical importance of addressing the needs of HNHC patient populations, there is surprisingly little data examining differences in spending and utilization in care for specific patient populations across countries. This hampers countries' ability to learn from one another or help them identify potential strategies to improve the care they offer to these patients.

To address this gap, we formed the International Collaborative on Costs, Outcomes, and Needs in Care (ICCONIC). This research collaborative included partners across 11 countries: Australia, Canada, England, France, Germany, the Netherlands, New Zealand, Spain, Sweden, Switzerland, and the United States. The goal was to carry out cross‐country comparisons of specific HNHC patient populations using a methodology that builds upon previous international comparisons work. The methodology employed the use of clinical case vignettes, similar to tracer conditions, which allowed for the systematic identification of similar groups of patients. Based on a HNHC typology recently put forward by the National Academy of Medicine,^6^ we identified two specific patient types or “personas”: a frail older adult who sustains a hip fracture with subsequent hip replacement or osteosynthesis and an older person with complex multimorbidity who is hospitalized with heart failure and has a comorbid diagnosis of diabetes.

This *Health Services Research* special issue highlights the key findings of the work stemming from the ICCONIC project, which include six original research articles that examine detailed variation in spending, utilization, and patient outcomes of the two personas across different components of the health system.^11^, ^12^, ^13^, ^14^, ^15^, ^16^, ^17^ In this perspective, we discuss the key findings of the ICCONIC project and how our work attempts to overcome major challenges that arise when conducting international comparisons across countries. We also discuss important lessons that policy makers may take away from this work in their efforts to improve the efficiency of their health systems.

## 2 KEY LESSONS FROM THE ICCONIC PROJECT

### 2.1 Use of patient vignettes helps overcome some issues of data comparability across countries

The ICCONIC project used a case‐vignette methodology to compare two specific types of HNHC personas across multiple care settings and countries constructed from patient‐level administrative and registry data. This approach is necessary to enable comparability across countries, given that national privacy regulations in most countries prohibit the broader sharing of patient‐level health data. Case vignettes, which describe specific groups of patients (based on age, gender, and diagnosis), allow for a clearer selection of similar patients across countries from which to construct descriptive estimates. The more detailed the case vignettes are, the more similar the patients being compared across countries will be. A handful of European projects have demonstrated the utility of using this approach to measure and compare spending and utilization across countries.^18^, ^19^, ^20^, ^21^ However, to date, these projects have mostly focused on selected European countries, and most were limited to comparisons of inpatient data.

Figueroa et al. highlights in detail how the collaborative identified and constructed the case‐vignettes for the two HNHC personas and reviews their comparability, highlighting that the cohorts are similar in age and sex composition.^11^ In addition, for the hip fracture persona, countries had relatively similar patterns of diagnostic codes and procedure codes. Given that the data across countries could not be pooled for analysis, we believe this method allows for improved like‐for‐like comparison of patients than available alternatives.

### 2.2 Comparisons of care trajectories reveal important insights, including potential substitution effects across different care settings

Most cross‐country comparisons to date have focused on looking at variations in the utilization and cost of hospital care specifically. One of the key results that emerged from the ICCONIC project was that there is considerable variability across health systems with regards to the relative share of care that occurs in hospitals relative to other care settings.^11^, ^12^ For example, our findings showed that for both personas, relative hospital utilization in the United States as compared to other countries was low; however, the United States had some of the highest utilization of rehab care days spent outside the hospital in short term rehab and skilled‐nursing facilities. When comparing total utilization, across both care settings, the United States went from being the lowest utilizer to among the highest. In contrast, in countries like Germany, significant time is spent in the hospital setting, given lack of an extensive out‐of‐hospital rehab infrastructure.

Potential substitution of care across settings extends across the entire care pathway. Our results also show that relative provision of outpatient services (primary care vs. outpatient specialty care) differs across countries, with the United States using relatively more specialty care than primary care relative to comparators. Long‐term care access across countries likely further influences these trends, although a lack comprehensive data on this type of care across countries did not allow us to explore this thoroughly. Across countries which had detailed data on post‐acute and long‐term care, there appeared to be some substitution between institutional‐ and community‐based (or home) rehab care.^15^

The location of care delivery has important implications for both costs and quality, particularly if you consider patient's preferences for spending time outside institutional settings. To better quantify these implications, more comparable data are required across all countries for the entire trajectory of care and on a broader set of outcomes such as patient‐reported outcome measures and quality of life. Future work on health systems comparison should further explore these differences and their implications for health system efficiency.

### 2.3 There are relatively similar patterns of spending and utilization by age and sex across countries

The ICCONIC findings show that there are differences of spending and utilization within countries by sex that are largely similar across countries.^14^ Women tended to spend more time in hospital during their index stay, as compared to men, although on average their expenditures were lower. Women also tended to use more rehabilitation services compared to men, both in hospital facilities and in the community, as well as more nursing care at home. Women, however, generally had fewer outpatient specialist visits as compared to men, possibly because they are more likely to be in a hospital or rehab setting throughout the year.

Similarly, our work found that end‐of‐life expenditures were lower for older patients as compared to their younger counterparts across most countries.^13^ This could be due to patient needs being met with lower spending, efficiency considerations, or the different management and allocation of the place of service delivery during the trajectory of care.

### 2.4 HNHC patients incur more resource use than their healthier counterparts, but where this resource use occurs differs by health system

A key objective of the ICCONIC project was to better understand the resource use a patient incurred by being a HNHC individual. To do this, we established a set of comparators for each of the two personas in the ICCONIC project that allowed us to estimate this excess resource use within and across countries. For the hip persona, we examined historic costs to understand how much an acute event such as a hip fracture changes the utilization and spending for an older frail adult.^17^ For the older adult with multimorbidity, we examined spending of different cohorts of patients to include subsets with limited comorbidity (CHF only) and greater comorbidity (CHF with diagnoses of diabetes and COPD).^12^

The historic data for the hip persona illustrated that utilization and costs for patients increased substantially in the year following a hip fracture. These increases were concentrated in the hospital sector across most countries, apart from the United States and Germany where they were greatest for postacute care spending. In some countries, the expenditure in primary care, outpatient specialty, and outpatient drugs declined in the year after the hip fracture, possibly because these patients were institutionalized in the inpatient and rehab setting for prolonged periods of time. Further, the comparison of patients with different levels of complex multimorbidity illustrated the relative increases in expenditures and utilization as complexity increases. While absolute increases in costs varied across countries, the relative increase was similar although there were some differences with regards to which care settings these additional costs were concentrated. More analysis of where costs are concentrated for different types of HNHC patients has the potential to reveal where integrated care, or the delivery of care in alternative settings, may yield cost savings for different systems.

### 2.5 Drivers of high spending differ across countries, with the United States having high prices per unit

Total expenditures of care can be driven by two main factors: utilization of care and the cost per unit. The ICCONIC data illustrate that there are important cross‐country differences in what drives variations in relative expenditures by setting. For example, when examining the inpatient expenditure data across both personas, Australia's expenditures were largely driven by a greater number of hospitalizations throughout the year, despite having a relatively low unit cost per hospitalization. The United States, on the other hand, had among the lowest number of hospitalizations but among the highest costs per hospitalization. In fact, across all settings, the United States had the highest prices per unit. High costs in the United States, however, were not just driven by higher prices. Importantly, we found that the United States had much higher utilization in the postacute care setting, namely skilled‐nursing facilities (SNFs), and also higher relative use of outpatient specialty care, which has a higher unit price per visit than primary care. Overall, these differences accounted for greater spending per patient in the United States relative to other countries.^12^, ^17^

### 2.6 There is substantial variation in health outcomes across countries, which does not appear to be correlated with health spending

The ICCONIC project collected information on mortality and readmissions for both personas at different intervals: 30, 180, and 365 days.^16^ Of note, England reported the highest mortality for both personas across all intervals. More investigation at the national level is needed to better understand what accounts for this mortality, which could be associated with a range of factors including differences in the data sample, care provision, and factors outside the health care system such as the provision of long‐term care.

Across the remaining countries, it was difficult to discern any clear consistent pattern in health outcomes across time intervals and persona. Most countries performed similarly to one another on average. However, countries spent considerably different amounts per patient over the course of a year and by setting. Our results suggest that these spending differences by and large did not correlate with the measures of health outcomes collected, for example, the highest spenders did not have the better outcomes.

### 2.7 Improvements in data access and data comparability are needed to carry out more robust health system comparisons in the future

The ICCONIC project sought to access individual‐level data across the care trajectory for a set of 11 high‐income countries. While all countries could provide relatively comparable inpatient data, countries were limited to the extent to which they could access comprehensive data across other care settings and the extent to which data from one setting could be linked to data in another. While many countries collect patient‐level data on most of the sectors of care being investigated, these are often held by different entities and require different authorization for access and approval. In some countries, the approval processes for accessing data constituted a major barrier for research use because of the time to obtain ethical approvals, the inability or lack of approval to link existing national datasets on different parts of the care trajectory for research purposes, and at times the cost of the data itself.

In addition, outside the inpatient setting, there were important differences with regards to the information standards and protocols used to collect, classify, and store data. Data collection was largely influenced by local incentive structures, such as payment models. For example, countries utilize different care facilities to deliver postacute care services. In Germany, patients received rehabilitative care largely in the inpatient setting, while in England, this occurred in many locations including the inpatient, outpatient, and social care settings. In the United States, postacute care services largely occurred in SNFs. In the Netherlands and Sweden, much of these services were provided in patient's homes. Under current measurement approaches, comparisons that focus on data from one care setting may not necessarily compare the same type of functional care.

To overcome this limitation and avoid producing misleading policy insights, it is important that international comparisons used more linked data that allow researchers to observe care across all sectors of the health system. Policy makers should consider how they can further enable data access to allow future comparative health systems research. In addition, more work needs to be carried out to create a taxonomy of health system utilization that corresponds to functional care received, rather than the setting in which care is received. The ICCONIC project identified and documented many of these differences, working together with national policy makers, clinicians, and coders to outlining important national differences across care delivery for the two HNHC personas being investigated.^11^ More work is needed to generate broader insights across other types of patients and identify a platform to share these for other researchers to build upon.

## 3 DISCUSSION AND CONCLUSIONS

The aims of the ICCONIC project were twofold: to explore the feasibly and potential of cross‐country comparisons of care trajectories and to examine the extent to which comparisons of specific HNHC patients across countries could provide national policy makers with insights for care improvement. Our findings illustrated that it is not only feasible but important to compare care trajectories across countries across countries using specific patient vignettes. Not exploring the full trajectory can lead to misleading conclusions about resource use and outcomes.

This work yields important insights for national policy makers with a frame of reference to assess their performance and identify areas for improvement or further exploration. For example, in England, more research is needed to understand what is driving higher mortality rates, and whether improved care delivery in the postacute phase has the potential to improve outcomes. However, for most countries, despite considerable variation in cost, outcomes for these populations are broadly similar. This suggests that there are potential opportunities for cost savings and efficiency gains across countries that should be further explored. One area where policy makers should focus on is around the setting in which care is delivered and further exploring whether care can be moved to lower cost settings without compromising outcomes. For example, one strategy in the United States is to create the infrastructure and incentives to shift more facility‐based rehab care to the home care setting with appropriate long‐term and home health care supports. Such a system exists currently in the Netherlands and in Sweden, which may explain why these two countries are arguably more efficient (achieve similar to better health outcomes as lower prices). Another area for consideration is to encourage relatively more use of primary care services over costlier outpatient specialty services for the management of chronic conditions like heart failure and diabetes, as is done by most countries in this study outside of the United States and Canada.

Our work shows how international comparisons of patient care trajectories across health systems are a useful tool to help national policy makers and clinical leaders understand how best to deliver care for complex patients and improve allocation of resources in countries that share similar populations and problems. As data access and comparability improve, further international comparative analyses will continue to be instrumental to improve the effectiveness and efficiency of health systems globally.

## References

[1] Berwick DM , Nolan TW , Whittington J . The triple aim: care, health, and cost. Health Aff. 2008;27(3):759‐769. 10.1377/hlthaff.27.3.759 18474969

[2] Bodenheimer T , Sinsky C . From triple to quadruple aim: care of the patient requires care of the provider. Ann Fam Med. 2014;12(6):573‐576. 10.1370/afm.1713 25384822 PMC4226781

[3] OECD . Health at a Glance 2017: OECD Indicators. OECD Publishing; 2017.

[4] Blumenthal D , Chernof B , Fulmer T , Lumpkin J , Selberg J . Caring for high‐need, high‐cost patients ‐ an urgent priority. N Engl J Med. 2016;375(10):909‐911. 10.1056/NEJMp1608511 27602661

[5] Figueroa JF , Jha AK . Approach for achieving effective care for high‐need patients. JAMA Intern Med. 2018;178(6):845‐846. 10.1001/jamainternmed.2018.0823 29630697

[6] Effective Care for High‐Need Patients . Opportunities for Improving Outcomes, Value, and Health. National Academy of Medicine. 2017. 37748007

[7] French E , Kelly E . Medical spending around the developed world. Fisc Stud. 2016;37(3–4):327‐344. 10.1111/j.1475-5890.2016.12127 PMC6680320 31404348

[8] Wodchis WP , Austin PC , Henry DA . A 3‐year study of high‐cost users of health care. CMAJ. 2016;188(3):182‐188. 10.1503/cmaj.150064 26755672 PMC4754179

[9] Tanke MAC , Feyman Y , Bernal‐Delgado E , et al. A challenge to all. A primer on inter‐country differences of high‐need, high‐cost patients. PLoS One. 2019;14(6):e0217353. 10.1371/journal.pone.0217353 31216286 PMC6583982

[10] Wieser S , Tomonaga Y , Riguzzi M , et al. Die Kosten der nichtübertragbaren Krankheiten in der Schweiz. Schlussbericht. 2014. 10.5167/uzh-103453

[11] Figueroa JF , Horneffer KE , Riley K , Abiona O , Arvin M , et al. A methodology for identifying high‐need, high‐cost patients for international comparisons. Health Serv Res. 2021. 10.1111/1475-6773.13890 PMC8579201 34755334

[12] Figueroa JF , Papanicolas I , Riley K , et al. International comparison of health spending and utilization among people with complex multimorbidity. Health Serv Res. 2021. 10.1111/1475-6773.13708 PMC8579210 34350586

[13] Blankart R , Van Gool K , Papanicolas I , et al. International comparison of spending and utilization at the end of life for hip fracture patients. Health Serv Res. 2021. 10.1111/1475-6773.13734 PMC8579204 34490633

[14] Or Z , Shatrov K , Penneau A , et al. Within‐country variations in healthcare consumption and spending among patients with heart failure and diabetes. Health Serv Res. 2021. 10.1111/1475-6773.13854 PMC8579197 34409601

[15] Wodchis WP , Or Z , Blankart CR , et al. An international comparison of long‐term care trajectories and spending following hip fracture. Health Serv Res. 2021. 10.1111/1475-6773.13864 PMC8579202 34378190

[16] Papanicolas I , Riley K , Abiona O , et al. Differences in health outcomes for high‐need high‐cost patients across high‐income countries. Health Serv Res. 2021. 10.1111/1475-6773.13735 PMC8579207 34378796

[17] Papanicolas I , Figueroa JF , Schoenfeld AJ , et al. Differences in health care spending and utilization among older frail adults in high‐income countries: ICCONIC hip fracture persona. Health Serv Res. 2021. 10.1111/1475-6773.13739 PMC8579209 34390254

[18] Häkkinen U , Iversen T , Peltola M , et al. Health care performance comparison using a disease‐based approach: the EuroHOPE project. Health Policy. 2013;112(1–2):100‐109. 10.1016/j.healthpol.2013.04.013 23680074

[19] Busse R , EuroDRG group . Do diagnosis‐related groups explain variations in hospital costs and length of stay? Analyses from the EuroDRG project for 10 episodes of care across 10 European countries. Health Econ. 2012;21(Suppl 2):1‐5. 10.1002/hec.2861 22815107

[20] Bernal‐Delgado E , Christiansen T , Bloor K , et al. ECHO: health care performance assessment in several European health systems. Eur J Pub Health. 2015;25(Suppl 1):3‐7. 10.1093/eurpub/cku219 25690123

[21] Schreyögg J , Stargardt T , Velasco‐Garrido M , Busse R . Defining the “health benefit basket” in nine European countries. Evidence from the European Union health BASKET project. Eur J Health Econ. 2005;6:2‐10. 10.1007/s10198-005-0312-3 16270212 PMC1388078

